# Effects of vitamin D supplementation on metabolic parameters, serum irisin and obesity values in women with subclinical hypothyroidism: a double-blind randomized controlled trial

**DOI:** 10.3389/fendo.2023.1306470

**Published:** 2023-12-21

**Authors:** Sara Safari, Maryam Rafraf, Mahsa Malekian, Roghayeh Molani-Gol, Mohammad Asghari-Jafarabadi, Majid Mobasseri

**Affiliations:** ^1^ Student Research Committee, Tabriz University of Medical Sciences, Tabriz, Iran; ^2^ Nutrition Research Center, Department of Community Nutrition, Faculty of Nutrition and Food Science, Tabriz University of Medical Sciences, Tabriz, Iran; ^3^ Endocrine Research Center, Tabriz University of Medical Sciences, Tabriz, Iran; ^4^ Cabrini Research, Cabrini Health, Malvern, VIC, Australia; ^5^ School of Public Health and Preventative Medicine, Faculty of Medicine, Nursing and Health Sciences, Monash University, Melbourne, VIC, Australia

**Keywords:** Vitamin D, subclinical hypothyroidism, TSH, metabolic parameters, irisin, obesity

## Abstract

**Purpose:**

Subclinical hypothyroidism is an early, mild form of hypothyroidism that may progress to overt hypothyroidism if untreated. The current study aimed to assess the effects of vitamin D supplementation on hormonal (thyroid stimulating hormone [TSH], triiodothyronine, thyroxine, and free thyroxine) parameters, lipid profiles, serum irisin, and obesity indices in women with subclinical hypothyroidism.

**Methods:**

The present randomized, double-blind, placebo-controlled clinical trial was carried out on 44 women with subclinical hypothyroidism. The participants were allocated to two groups (22 patients in each group) that received vitamin D (50,000 IU/week) or placebo for 12 weeks. Fasting blood samples, anthropometric and body composition measurements, physical activity levels, and dietary intakes were collected at baseline and at the end of the study.

**Results:**

Vitamin D supplementation significantly decreased TSH, total cholesterol, and fat mass percentage, and significantly increased serum vitamin D and irisin levels and fat-free mass percentage compared to the control group (all, p<0.05). Changes in thyroid hormones, other lipid profiles, and anthropometric indices were not significantly different between the groups.

**Conclusion:**

Our study indicates that vitamin D administration improves serum TSH, total cholesterol, irisin, and body composition in women with subclinical hypothyroidism. More well-designed clinical trials are required to confirm these findings and clarify the effects of vitamin D supplementation on both genders of patients.

**Clinical trial registration**

https://www.irct.ir/trial/57482, Identifier IRCT20100408003664N25.

## Introduction

Thyroid diseases are one of the most common endocrine disorders in the world (1), affecting approximately 40% of the world’s population (2). Thyroid hormones, including triiodothyronine (T_3_) and thyroxine (T_4_), have prominent effects on human metabolism, growth, and development. Moreover, they have profound metabolic effects in adult life, including maintaining thermogenic and metabolic homeostasis, reducing body weight, and affecting muscle function (3, 4).

There are numerous thyroid disorders. Hypothyroidism is a pathophysiological phase with insufficient hormone production, resulting in an imbalance in basal metabolic rate and inefficient physiological roles of the body systems (1). Hypothyroidism might be clinical/overt, with an elevation in the thyroid-stimulating-hormone (TSH) and low levels of free T_4_ (fT_4_), or subclinical, with normal levels of fT_4_ and elevated serum TSH (5).

The prevalence of subclinical hypothyroidism in adults is 4–20%, depending on such factors as race, age, and ethnicity. Subclinical hypothyroidism has various causes, including chronic autoimmune thyroiditis, iodine deficiency, etc. The disorder is related to antithyroid peroxidase antibodies in about 60–80% of cases. Subclinical hypothyroidism patients have few signs and symptoms. The presence of antithyroid peroxidase antibodies and female gender are linked with an enhanced risk of progression to overt hypothyroidism. Those with TSH levels higher than 10 mIU/L should be treated, and L-thyroxine is the common treatment in overt hypothyroidism (6).

Vitamin D is a fat-soluble vitamin effective in reducing the risk of chronic diseases, such as bone metabolic disorders, type 2 diabetes mellitus (T2DM), cardiovascular diseases, autoimmune diseases, and hypothyroidism (7–9). Vitamin D mediates its influence via binding to the vitamin D receptor (VDR) and activating the gene response in different cells such as thyroid follicular cells (10). There is a strong homology between the VDR molecular structure and the thyroid hormone receptor (11).

According to the results of some studies, subjects with subclinical hypothyroidism have a lower serum 25-hydroxyvitamin D3 (25-OH D3) than normal limits, and vitamin D supplementation improves thyroid function by reducing TSH in these patients (12–14). Some other studies reported that vitamin D supplementation may affect metabolic status and obesity indexes in different diseases such as T2DM and atherosclerosis (15, 16). The results of a retrospective cohort study by Alsamghan et al. on 400 hypothyroid patients showed an inverse relationship between 25-OH D3 and dyslipidemia (17). However, Anaraki et al. investigated 65 vitamin D-deficient euthyroid or hypothyroid patients for 12 weeks and reported that using 50,000 IU oral vitamin D3 supplementation per week did not improve weight or body mass index (BMI) in hypothyroid patients (18).

Irisin is a hormone mainly synthesized by skeletal muscle and fat tissue. The mRNA of the fibronectin type III domain-containing protein 5 (FNDC5) gene precursor of irisin is found in various tissues, including the thyroid gland and fat tissue. Many researchers have reported its significant effect on metabolism and thermogenesis. Therefore, thyroid function can be directly or indirectly related to the regulation of irisin or, conversely, irisin can affect the thyroid. It was also reported that vitamin D supplementation may affect serum irisin concentrations in subjects with vitamin D deficiency and in T2DM patients (19, 20). Nevertheless, no study has investigated the possible effects of vitamin D supplementation on serum irisin levels in patients with hypothyroid dysfunction such as subclinical hypothyroidism.

Although vitamin D deficiency has been reported mainly in patients with subclinical hypothyroidism (12–14), limited studies have evaluated its supplementation effects on metabolic parameters. Moreover, its possible effects on obesity indices and serum irisin levels have not been investigated in these patients so far. Accordingly, this study aimed to assess the effect of vitamin D supplementation on TSH and thyroid hormones, lipid profiles, serum irisin levels, and obesity indices in women with subclinical hypothyroidism.

## Materials and methods

### Participants

A total of 44 women with subclinical hypothyroidism were included in this double-blind, randomized, placebo-controlled trial. Patients were enrolled at the endocrinology clinic of Tabriz, Iran, from September 2021 to June 2022. The inclusion criteria were females aged 20–65 years old; diagnosed with subclinical hypothyroidism, TSH 5–10 µIU/mL, normal fT_4_, BMI of 25–37 kg/m^2^, and willingness to participate. Exclusion criteria were pregnancy and breastfeeding; having T2DM, hypertension, cardiovascular, kidney, liver, and autoimmune diseases; serum vitamin D levels above 100 ng/ml; use of any kind of medications; history of vitamin D supplement or any kind of supplement consumption during the study period and last 2 months before the intervention; smoking and use of alcohol. The protocol of this clinical trial was approved by the Ethics Committee of the Tabriz University of Medical Sciences (Registration number: IR.TBZMED.REC.1400.327) and it was also registered in the Iranian Registry of Clinical Trials (IRCT registration number: IRCT20100408003664N25). The research was performed in accordance with the Declaration of Helsinki principles.

### Study design

Participants were randomly allocated to the vitamin D supplement (n=22) or the placebo (n=22) groups utilizing a randomized block of size 4. A list of random numbers generated by Random Allocation Software (RAS) was used to allocate the participants. Researchers and patients were blinded to the treatments’ allocation. A general questionnaire was completed for all of the participants at the beginning of the study. The vitamin D group (n =22) received 50,000 IU/week of vitamin D and the control group (n =22) received a placebo containing sunflower oil for 12 weeks. Sunflower oil is one of the sources of vitamin E. However, the amount of vitamin E in one placebo pearl is negligible because the amount of vitamin E in one tablespoon of sunflower oil is 5.75 mg (21). Both vitamin D and placebo pearls that were provided by Zahravi Pharmaceutical Company (Zahravi Pharmaceutical Co, Tabriz, Iran) were administered once a week after lunch. The appearance of the vitamin D supplement and placebo were identical. It was recommended that all of the participants maintain their usual dietary habits, sunlight exposure, and physical activity all over the study. A follow-up visit was carried out every 6 weeks, and the participants’ compliance with the study procedure was tracked by recursive pearls. In addition, participants were assessed for any possible complications at each follow-up visit.

### Measurements

Anthropometric measures were obtained at baseline and the end of the trial. The body weight was measured without shoes and in minimal clothing by a Seca scale (Seca, Hamburg, Germany) nearest to 0.1 kg. Height was also recorded without shoes by wall-mounted tape nearest to 0.5 cm. BMI was calculated as the weight in kg divided by the square of height in meters. Waist circumference (WC) was measured using a tape at the midpoint between the lowest rib and the superior border of the iliac crest and was approximately recorded at 0.5 cm. Body composition (fat-free mass (FFM) and fat mass (FM)) was measured by bioelectrical impedance analysis method using Tanita body composition analyzer. Dietary intake data were collected using a three-day food record (1 weekend day and 2 weekdays) at baseline and the end of the study. Then, the participants’ diets were assessed using Nutritionist IV software (First Databank Inc, Hearst Corp, San Bruno, CA), and dietary energy, carbohydrate, fat, protein, and vitamin D intakes were obtained. Moreover, a short and valid form of the International Physical Activity Questionnaire was applied to evaluate the physical activity level of each participant at baseline and the end of the trial (22).

Blood samples (5 ml) were obtained from each participant’s antecubital vein after 12 hours of overnight fasting at baseline and the end of the trial. After centrifugation of the blood, serum samples were frozen at -70^°C^ until the assay time. 25-OH D3 levels were measured by using ELISA kits (Monobind kit, Monobind Inc., Lake Forest, CA, USA). TSH was measured using commercial ELISA kits (TSH, Pishtaz Teb Zaman Diagnostics, Tehran, Iran) based on the instructions of the manufacturer. The concentration of T_4_, T_3_, and fT_4_ was determined using an ELISA kit (Pishtaz Teb Zaman Diagnostics, Tehran, Iran). Triglyceride (TG), total cholesterol (TC), and HDL-C were measured by standard enzymatic method with commercial kits (Pars Azmoon, Tehran, Iran). Friedewald formula was applied for low-density lipoprotein cholesterol (LDL-C) calculation: LDL-C = TC (mg/dl) _ ([HDL-C (mg/dl) + TG (mg/dl)]/5). The ELISA method with the human irisin ELISA kit E3253Hu (Bioassay Technology- China) was used to measure the serum concentration of irisin. The intra- and inter-assay coefficients of variation for irisin were <8% and <10%, respectively, and the sensitivity was 0.095 ng/ml.

The primary outcome includes metabolic parameters (serum level of vitamin D, thyroid hormones, lipids profile, and irisin) and the secondary outcomes were the measurements of obesity indices and body composition.

### Statistical analysis

Statistical analyses were done using SPSS software (version 16; SPSS Inc, Chicago, IL). The normality of the data distribution was assessed by the Kolmogorov–Smirnov test and descriptive indices. The results of quantitative data with normal and non-normal distribution are reported as mean ± SD and median (25th and 75th percentiles), respectively. Frequency (percent) is reported for qualitative data. Due to the non-normal distribution of irisin, logarithmic transformation was performed before the analysis. Comparison of baseline characteristics between the groups was done using the independent sample t-test, Mann–Whitney U test, and chi-squared test. To compare within-group changes before and after the intervention, the paired sample t-test and Wilcoxon signed-rank test were applied. The changes between the two groups after the intervention were compared by analysis of covariance adjusting for the baseline values and other potential confounders. Each variable’s percentage change was obtained by the following formula: [(after mean value - before mean value)/before mean value] 100. All analyses were carried out by the intention-to-treat principle, and *p*-values less than 0.05 (*p* < 0.05) were considered statistically significant.

## Results

### General characteristics

**Figure 1:**
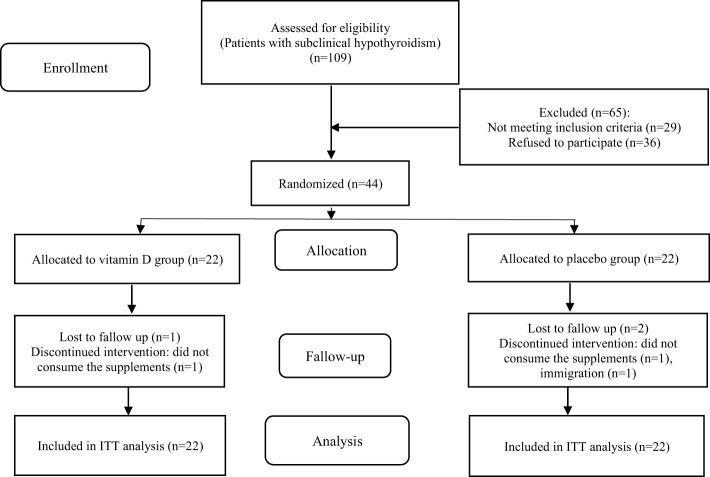
Study flow diagram. ITT, Intention-to-treat.

Forty-four women with subclinical hypothyroidism were enrolled in the trial and 41 participants (21 in the vitamin D group and 20 in the placebo group) completed the 12-week study. The reasons for withdrawal from the intervention are outlined in the study flow diagram (**Figure 1**). The compliance with the study protocol based on counting the number of recursive pearls was more than 96% among participants who completed the trial. Throughout the study, no adverse effects were reported.

The baseline characteristics of the patients are presented in **Table 1**. The age, duration of casual sunlight exposure, and physical activity levels of patients were not significantly different between the two groups at baseline. No significant changes were observed in sunlight exposure and physical activity levels of participants in either group during the study period (*p*>0.05).

**Table 1:** Baseline characteristics of the study participants.

Variables	Placebo group (n=22)	Vitamin D group (n=22)	*p* value
**Age (year)**	36.050 ± 11.125	36.360 ± 11.676	0.927^a^
**Duration of casual sunlight exposure (min/day)**	20.000 (10.000, 45.000)	20.000 (8.00, 32.500)	0.661^b^
**PAL, n (%)** ** Light** ** Moderate** ** Vigorous**	9 (40.909) 13 (59.090) 0 (0.000)	10 (45.454) 11 (50.000) 1 (4.545)	0.544^c^

PAL, physical activity level.

Mean ± SD and Median (percentile 25 and 75) are presented for normally and not normally distributed measures, respectively.

a Independent sample t-test.

b Mann–Whitney U test.

c Chi-square.

### Dietary intake

**Table 2** presents the dietary intake data of the participants. No significant differences were observed in energy, carbohydrate, fat, and vitamin D intakes between the two groups at baseline. At the beginning of the trial, there was a significant difference in protein intake between the two groups (*p* = 0.018). Changes in total energy, macronutrients, and vitamin D intakes were not significant within the groups during the study (*p*>0.05). Based on the analysis of covariance adjusted for baseline values, no significant differences were observed in the mean of energy, macronutrients, and vitamin D intakes between the vitamin D and placebo groups at the end of the study (*p* > 0.05).

**Table 2:** Daily dietary intake of participants at baseline and 12 weeks after the intervention.

Variables	Placebo group (n=22)	Vitamin D group (n=22)	MD (CI 95%)	*p* value
**Total energy, kcal/day** ** Before** ** After** ** *p* value**	1816.148 ± 231.057 1850.585 ± 251.212 0.468^c^	1711.188 ± 227.153 1751.028 ± 284.776 0.308^c^	-104.960 (-244.370, 34.449) -99.557 (-262.944, 63.830)	0.136^a^ 0.809^b^
**Carbohydrate, (g)** ** Before** ** After** ** *p* value**	237.405 ± 43.309 250.831 ± 43.801 0.164^c^	225.344 ± 35.386 242.203 ± 40.249 0.113^c^	-12.061 (-36.124, 12.001) -8.628 (-34.222, 16.966)	0.318^a^ 0.751^b^
**Protein, (g)** ** Before** ** After** ** *p* value**	69.419 ± 14.697 67.968 ± 13.874 0.657^c^	59.280 ± 12.107 60.794 ± 15.755 0.672^c^	-9.683 (-17.626, -1.740) -7.173 (-16.206, 1.859)	0.018^a*^ 0.516^b^
**Fat, (g)** ** Before** ** After** ** *p* value**	67.425 ± 9.930 66.381 ± 12.946 0.653^c^	64.975 ± 18.683 60.376 ± 16.682 0.106^c^	-2.450 (-11.553, 6.653) -6.004 (-15.089, 3.080)	0.590^a^ 0.200^b^
**Vitamin D intake, (µg)** ** Before** ** After** ** *p* value**	0.626 ± 0.511 0.635 ± 0.603 0.963^c^	0.658 ± 0.498 0.646 ± 0.621 0.938^c^	0.031 (-0.276, 0.338) 0.010 (-0.361, 0.383)	0.839^a^ 0.960^b^

MD (%95 CI); mean difference (%95 confidence interval). Data are expressed as mean ± SD.

a Independent sample t-test.

b Analysis of covariance (ANCOVA) adjusted for baseline values.

c paired sample t-test.

^∗^p value ≤ 0.05.

The bold values are statistically significant.

### Biochemical parameters

The biochemical parameters of the patients at baseline and the end of the trial are shown in **Table 3**. All of the biochemical values were not significantly different between the two groups at baseline (*p*>0.05). Serum TSH and TC significantly decreased in the vitamin D group in comparison with the baseline values (by 20.54%, *p*<0.001 and 3.44%, *p*<0.001, respectively). 25-OH D3 levels significantly increased in the vitamin D group in comparison with the baseline measurements (by 98.59%, *p*<0.001). Changes in other variables within the vitamin D group were not significant. Within-group changes for all variables were not significant in the placebo group.

**Table 3:** Biochemical characteristics of participants at baseline and 12 weeks after the intervention.

Variables	Placebo group (n=22)	Vitamin D group (n=22)	MD (CI 95%)	*p* value
**Vitamin D, (ng/ml)** **Before** **After** **MD (CI 95%)** ***p* value**	24.700 ± 9.672 26.664 ± 9.143 1.964 (-0.440, 4.369) 0.104^c^	22.221 ± 7.794 44.129 ± 8.820 21.907 (18.269, 25.545) <0.001^c*^	-2.478 (-7.823, 2.865) 19.827 (15.696, 23.957)	0.355^a^ <0.001^b*^
**TSH, (µIU/mL)** **Before** **After** **MD (CI 95%)** ***p* value**	7.272 ± 1.455 7.014 ± 1.243 -0.258 (-0.728, 0.211) 0.266^c^	7.150 ± 1.107 5.681 ± 1.003 -1.468 (-1.685, -1.251) <0.001^c*^	-0.122 (-0.909, 0.664) -1.332 (-2.021, -0.644)	0.755^a^ <0.001^b*^
**T_3_, (ng/ml)** **Before** **After** **MD (CI 95%)** ***p* value**	1.805 ± 0.277 1.809 ± 0.150 0.004 (-0.138, 0.145) 0.958^c^	1.810 ± 0.232 1.827 ± 0.216 0.017 (-0.113, 0.148) 0.787^c^	0.004 (-0.151, 0.160) 0.018 (-0.095, 0.131)	0.953^a^ 0.996^b^
**T_4_, µ)g/dl)** **Before** **After** **MD (CI 95%)** ***p* value**	8.186 ± 1.227 8.192 ± 1.022 0.006 (-0.602, 0.614) 0.230^c^	7.927 ± 1.238 8.214 ± 1.326 0.287 (-0.347, 0.921) 0.082^c^	-0.259 (-1.009, 0.491) 0.021 (-0.439, 0.919)	0.490^a^ 0.751^b^
**fT_4_, (ng/dl)** **Before** **After** **MD (CI 95%)** ***p* value**	1.204 ± 0.096 1.212 ± 0.123 0.007 (-0.037, 0.051) 0.739^c^	1.214 ± 0.075 1.255 ± 0.094 0.041 (-0.003, 0.085) 0.066^c^	0.009 (-0.043, 0.062) 0.043 (-0.023, 0.110)	0.716^a^ 0.488^b^
**TC, mg/dl** **Before** **After** **MD (CI 95%)** ***p* value**	172.318 ± 22.015 171.745 ± 18.433 -0.572 (-4.711, 3.565) 0.760^c^	176.590 ± 25.216 170.510 ± 23.025 -6.080 (-8.092, -4.089) <0.001^c*^	4.272 (-10.130, 18.675) -4.187 (-8.229, -0.144)	0.553^a^ 0.043^b*^
**TG, mg/dl** **Before** **After** **MD (CI 95%)** ***p* value**	86.909 ± 24.025 83.100 ± 21.727 -3.809 (-10.959, 3.341) 0.280^c^	89.772 ± 27.006 84.700 ± 29.668 -5.072 (-13.40, 3.257) 0.219^c^	2.863 (-12.688, 18.415) 1.600 (-14.222, 17.422)	0.712^a^ 0.857^b^
**HDL-C, mg/dl** **Before** **After** **MD (CI 95%)** ***p* value**	38.272 ± 7.132 38.700 ± 5.976 0.427 (-1.426, 2.280) 0.637^c^	37.500 ± 8.410 38.600 ± 8.138 1.100 (-0.498, 2.698) 0.167^c^	-0.772 (-5.517, 3.972) -0.100 (-4.444, 4.244)	0.744^a^ 0.447^b^
**LDL-C, mg/dl** **Before** **After** **MD (CI 95%)** ***p* value**	116.663 ± 22.604 115.704 ± 18.705 -0.959 (-5.708, 3.790) 0.679^c^	115.790 ± 35.013 107.173 ± 23.499 -8.617 (-19.627, 2.392) 0.119^c^	-0.872 (-18.804,17.058) -8.530 (-21.453, 4.392)	0.922^a^ 0.058^b^
**Irisin, (ng/ml) ^&^** **Before** **After** **MD (CI 95%)** ***p* value**	13.384 (12.200, 13.025) 12.918 (11.675, 13.362) -0.466 (3.48%) 0.122^e^	12.917 (11.825, 13.475) 14.175 (12.475, 14.975) 1.258 (9.74%) 0.054^e^	-0.466 (-1.819, 0.886) 1.546 (0.315, 2.778)	0.418^d^ 0.011^b*^

fT_4_, free T_4_; HDL-C, high-density lipoprotein cholesterol; LDL-C, low-density lipoprotein cholesterol; MD (%95 CI), mean difference (%95 confidence interval); TC, total cholesterol; TG, triglyceride; TSH, thyroid stimulating hormone; T_3_, triiodothyronine; T_4_, thyroxine.

Mean ± SD and Median (percentile 25 and 75) are presented for normally and not normally distributed measures, respectively. Differences of medians and change percent are presented for measures not normally distributed. Analysis after log transformation.

a Independent sample t-test.

b Analysis of covariance (ANCOVA) adjusted for baseline values, energy intake and physical activity.

c Paired samples t-test.

d Mann–Whitney.

e Wilcoxon.

^&^Analyzed after log-transformation.

^∗^p value ≤ 0.05.

The bold values are statistically significant.

According to the analysis of covariance adjusted for energy intake, physical activity, and baseline values, significant differences in serum levels of 25-OH D3 (MD (CI 95%): 15.266 (9.311, 21.221), *p*<0.001), TSH (MD (CI 95%): -1.332 µIU/mL (-2.021, -0.644), p<0.001), TC (MD (CI 95%): -4.187 mg/dl (-8.229, -0.144), *p*<0.001), and irisin (MD (CI 95%): 1.546 ng/ml (0.315, 2.778), *p*=0.011) were observed between two groups at the end of study. Changes in serum levels of T_3_, T_4_, fT_4,_ TG, HDL-C, and LDL-C between the two groups were not significant after the intervention (*p*>0.05).

### Anthropometric measurements

The anthropometric characteristics of the participants are shown in **Table 4**. At the beginning of the study, weight, BMI, WC, FM %, and FFM % of participants were not significantly different between the two groups (*p*>0.05). Within-group changes in all of the variables were not significant at the end of the study in any of the groups (*p*>0.05). Based on analysis of covariance adjusted for energy intake, physical activity, and baseline values, significant differences were observed in FM % (MD (CI 95%): -1.344% (-2.501, -0.186), p=0.024) and FFM % (MD (CI 95%): 1.897% (0.375, 3.419), p=0.016) between two groups at the end of the study. Changes in weight, BMI, and WC were not significantly different between the two groups (*p*>0.05).

**Table 4:** Anthropometric characteristics of participants at baseline and 12 weeks after the intervention.

Variables	Placebo group (n=22)	Vitamin D group (n=22)	MD (CI 95%)	*p* value
**Weight, kg** **Before** **After** **MD (CI 95%)** ***p* value**	74.454 ± 10.352 74.000 ± 10.046 -0.454 (-1.400, -0.491) 0.329^c^	74.688 ± 10.770 73.430 ± 10.374 -1.258 (-2.788, 0.271) 0.102^c^	0.234 (-6.193, 6.662) -0.570 (-6.783, 5.643)	0.942^a^ 0.431^b^
**BMI, kg/m^2^** **Before** **After** **MD (CI 95%)** ***p* value**	29.686 ± 2.573 29.689 ± 2.708 0.003 (-0.209, 0.216) 0.973^c^	29.589 ± 3.154 29.332 ± 3.038 -0.257 (-0.566, 0.518) 0.098^c^	-0.097 (-1.849, 1.654) -0.357 (-2.109, 1.393)	0.911^a^ 0.096^b^
**WC, cm** **Before** **After** **MD (CI 95%)** ***p* value**	90.090 ± 9.466 89.630 ± 9.195 -0.460 (-1.325, 0.403) 0.280^c^	93.640 ± 9.874 93.177 ± 9.836 -0.459 (-1.064, 0.146) 0.130^c^	3.545 (-2.340, 9.431) 3.547 (-2.246, 9.340)	0.231^a^ 0.844^b^
**FM, %** **Before** **After** **MD (CI 95%)** ***p* value**	33.977 ± 4.947 34.000 ± 4.876 0.022 (-0.824, 0.870) 0.956^c^	33.540 ± 6.106 32.714 ± 5.857 -0.826 (-1.776, 0.123) 0.085^c^	-0.436 (-3.818, 2.944) -1.344 (-2.501, -0.186)	0.796^a^ 0.024^b*^
**FFM, %** **Before** **After** **MD (CI 95%)** ***p* value**	63.469 ± 6.218 63.006 ± 6.576 -0.463 (-1.421, 0.495) 0.326^c^	64.956 ± 7.017 65.994 ± 6.908 1.039 (-0.131, 2.211) 0.079^c^	1.487 (-2.547, 5.521) 1.897 (0.375, 3.419)	0.461^a^ 0.016^b*^

BMI, body mass index; FFM, fat-free mass; FM, fat mass; MD (%95 CI), mean difference (%95 confidence interval); WC, waist circumference.

Data are expressed as mean ± SD.

a Independent sample t-test.

b Analysis of covariance (ANCOVA) adjusted for baseline values, energy intake and physical activity.

c Paired samples t-test.

^∗^p value ≤ 0.05.

The bold values are statistically significant.

## Discussion

Subclinical hypothyroidism is often considered an early sign of thyroid failure (6). It has been suggested that vitamin D may have a positive effect on thyroid function and reduce the likelihood of overt hypothyroidism (13). However, few studies are available about the effects of vitamin D supplementation on subclinical hypothyroidism.

According to our results, the mean 25-OH D3 level was lower than normal limits (≥30 ng/ml) (23) in both groups at the beginning of the study. Vitamin D supplementation increased 25-OH D3 levels and lowered TSH. As expected, T_3_, T_4_, and fT_4_ levels did not change significantly due to their normal levels in patients with subclinical hypothyroidism. These findings are in agreement with some previous studies on patients with subclinical hypothyroidism and vitamin D deficiency (12–14), as well as patients with hypothyroidism (15, 24, 25). VDR and TSH receptors have similar structures (11), and vitamin D modulates pituitary TSH secretion by binding to specific binding sites. Also, vitamin D can directly influence thyrocytes by attenuating the uptake of thyrotropin-stimulated iodide and cell growth (26).

In the current study, in addition to the reduction of TC levels, the lowering effects of vitamin D supplementation were considerable on LDL-C levels. In the study by Ucan et al., 50,000 IU/week of 25-OH-D3 intramuscularly for eight weeks lowered LDL-C after two months in patients with Hashimoto’s thyroiditis (25). The results of a meta-analysis that enrolled 17 studies confirmed the significant impact of vitamin D supplementation on reducing serum levels of TC and LDL-C in patients with T2DM, though changes in serum TG and HDL-C were negligible (27). On the other hand, in a study by Anaraki et al. on euthyroid or hypothyroid patients with vitamin D deficiency, TC did not change significantly after vitamin D supplementation (18). Bile acid synthesis, hepatocyte functions, and serum TC are affected by VDR, possibly via regulation of the expression of genes that have a role in bile acid synthesis from cholesterol (28). Studies on mouse and human hepatoma cells also revealed that VDR activation blocked farnesoid X receptor expression, thereby suppressing cholesterol 7α-hydroxylase expression and decreasing cholesterol levels (29, 30). In addition, Defay et al. showed that hydroxylated vitamin D derivatives inhibit the activity of 3-hydroxy-3-methylglutaryl coenzyme-A (HMG-CoA) reductases, which catalyzes 7-dehydrocholesterol to cholesterol (31). Moreover, vitamin D regulates the metabolism of calcium and increases intestinal calcium absorption, thereby decreasing intestinal fatty acid absorption. Therefore, averting intestinal fat absorption could lower the TC levels (32).

Our results indicate the potential effect of vitamin D supplementation on reducing TC levels. This effect might be mediated partly through the lowered TSH level determined in our intervention group. TSH can decrease phosphorylated HMG-CoA reductase expression via AMPK in the liver, leading to increased HMG-CoA reductase activity and hypercholesterolemia (33). In the present study, while the mean TG level was within the normal range at baseline, HDL-C was lower than normal (the cut-off value for HDL-C is 50 mg/dl for women) (34). Vitamin D’s lack of affecting TG and HDL-C levels may be because of homeostatic controlling mechanisms. The exact effects of vitamin D on HDL-C remain elusive (35–38), and further studies are needed.

According to our findings, vitamin D supplementation increased the serum levels of irisin. In the study by Al-Daghri et al. on 78 subjects with vitamin D deficiency who received adequate sunlight and ate vitamin D-enriched foods, irisin levels increased with vitamin D levels (19). Previous *in-vitro* studies on human endothelial cells proposed that vitamin D can promote the activation of the silent information regulator sirtuin 1 (SIRT1) and AMP-activated protein kinase (AMPK) in skeletal muscle cells. SIRT1 plays an important role in AMPK activation. AMPK and SIRT1 influence the activation and transcription of peroxisome proliferator-activated receptor gamma coactivator 1-alpha (Pgc1α) (39). Pgc1α is a co-transcriptional regulator that facilitates multiple transcription factors, regulating a complex network of genes; it also increases irisin, followed by increasing fibroblast growth factor 21 and uncoupling protein 1 (UCP1) (20, 40). Consistently, Pgc1α activation enhances the expression of irisin precursor FNDC5. Irisin is involved in both tissue mitochondrial content control and brown adipose tissue formation. Irisin activates mitochondrial UCP1, which inhibits ATP synthesis and augments thermogenesis by stimulating browning in white adipose tissues (41). All the mentioned factors contribute to body weight control. Irisin may also have a potential therapeutic role in muscular dystrophy (42).

Based on our findings, the elevated serum irisin levels in the vitamin D-supplemented group might be partly involved in the changes in FM % and FFM %. However, no considerable changes were seen in overall and abdominal obesity values, though a longer intervention period for maintaining sufficient vitamin D status might be required to obtain considerable reductions in obesity values. Similarly, in the study by Talaei et al., no significant alterations were observed in the weight and BMI of hypothyroid patients after receiving 50,000 IU/week of vitamin D for three months (15). Vitamin D supplementation with dissimilar doses and durations in other metabolic disorders also resulted in diverse outcomes (43–45). It has been proposed that 1,25-OH-D3 can inhibit adipogenesis through VDRs and promote and induce apoptosis in adipocytes (46). Insufficient 25-OH D3 levels may stimulate pre-adipocyte differentiation into adipocytes (47). Moreover, a direct consequence of low 25-OH D3 levels is an increment in parathyroid hormone, which increases calcium influx into adipocytes and subsequently promotes lipogenesis, resulting in considerable fat accumulation and weight gain (48).

It should be noted that in the current study, we did not intervene to make changes in dietary intakes, physical activity levels, and sunshine exposure time in either group during the study. Therefore, these factors were not accounted for as confounding factors in the interpretation of the biochemical and anthropometric variables.

The strength of this study is that it was a well-designed controlled clinical trial, eliminating potential sources of bias. However, some limitations existed, including the short study duration, small sample size, and fixed dose of vitamin D. Therefore, the findings of this study may not be generalized to other doses and durations of vitamin D supplementation. Also, our findings are not applicable to patients with low or normal BMIs or men with subclinical hypothyroidism. Further studies are needed to investigate the impact of vitamin D on SIRT1 and adiposity as well as on muscle strength via its effect on irisin levels.

## Conclusions

Our study indicates that vitamin D administration at a dose of 50,000 IU/week for 12 weeks decreases serum TSH and TC levels and elevates irisin levels in women with subclinical hypothyroidism without significantly affecting other lipid profile parameters. Moreover, vitamin D supplementation improved body composition in these patients. Hence, the consumption of vitamin D might be a helpful approach to control subclinical hypothyroidism in women. Nevertheless, more well-designed clinical trials are warranted to confirm these findings and to clarify the vitamin D supplementation effects on both genders of patients.

## Data availability statement

The data analyzed in this study is subject to the following licenses/restrictions: Data are available from the corresponding author upon reasonable request. Requests to access these datasets should be directed to MR, rafrafm@tbzmed.ac.ir.

## Ethics statement

The studies involving humans were approved by The Ethics Committee of Tabriz University of Medical Science (Registration number: IR.TBZMED.REC.1400.327). The studies were conducted in accordance with the local legislation and institutional requirements. Written informed consent for participation in this study was provided by the participants’ legal guardians/next of kin.

## Author contributions

SS: Data curation, Methodology, Writing – original draft. MR: Conceptualization, Supervision, Writing – review & editing. MMa: Data curation, Writing – original draft. RM-G: Methodology, Writing – review & editing. MA-J: Formal analysis, Writing – original draft. MMo: Data curation, Writing – review & editing.
